# Leptospirosis in febrile patients with suspected diagnosis of dengue fever

**DOI:** 10.1186/s13104-021-05627-3

**Published:** 2021-05-29

**Authors:** Juana del Valle-Mendoza, Carlos Palomares-Reyes, Hugo Carrillo-Ng, Yordi Tarazona-Castro, Sungmin Kym, Miguel Angel Aguilar-Luis, Luis J. del Valle, Ronald Aquino-Ortega, Johanna Martins-Luna, Isaac Peña-Tuesta, Eduardo Verne, Wilmer Silva-Caso

**Affiliations:** 1 School of Medicine, Research and Innovation Center of the Faculty of Health Sciences, Universidad Peruana de Ciencias Aplicadas, Lima, Peru; 2 Laboratorio de Biologia Molecular, Instituto de Investigación Nutricional, Lima, Peru; 3 Facultad de Medicina Alberto Hurtado, Universidad Peruana Cayetano Heredia, Lima, Perú; 4 Division of Infectious Disease, Department of Internal Medicine, Chungnam National University School of Medicine , Daejeon, Korea; 5 Barcelona Research Center for Multiscale Science and Engineering, Departament D’Enginyeria Química, EEBE, Universitat Politècnica de Catalunya (UPC), Barcelona, Spain

**Keywords:** Acute febrile illnesses, Leptospirosis, DENV, Tropical disease

## Abstract

**Objective:**

This study was carried out to determine the prevalence of leptospirosis among febrile patients with a suspicious clinical diagnosis of dengue fever in northern Peru.

**Results:**

A total of 276 serum samples from patients with acute febrile illness (AFI) and suspected diagnosis for dengue virus (DENV) were analyzed. We identified an etiological agent in 121 (47.5%) patients, DENV was detected in 30.4% of the cases, leptospirosis in 11.2% and co-infection by both pathogens was observed in 5.9% of the patients. In this study the most common clinical symptoms reported by the patients were: headache 89.1%, myalgias 86.9% and arthralgias 82.9%. No differences in symptomatology was observed among the different study groups.

## Introduction

Leptospirosis is a tropical disease caused by spirochetes of the genus *Leptospira* spp. [1]. It is a zoonotic and waterborne disease that is endemic in many developing countries [1, 2]. Its transmission occurs by direct or indirect exposure to urine of infected reservoir host animals [2, 3]. Leptospirosis is considered the most neglected tropical disease and the leading zoonotic cause of morbidity and mortality [2, 4], accounting for 1.03 million human cases every year and 58,000 deaths worldwide [4, 5]. Dengue fever is an arboviral disease caused by the infection of any of the four dengue virus serotypes, denominated as DENV 1–4 [6, 7]. It is the most important mosquito-borne disease worldwide, which is transmitted by the *Aedes aegypti* mosquito [6]. This virus is responsible for approximately 390 million infections every year and 25,000 deaths worldwide, affecting particularly tropical and subtropical regions [8, 9]. Both pathogens are common causes of acute febrile illnesses (AFI) in many developing countries [10, 11] and have the potential to cause large outbreaks due to heavy rainfall and flooding [11]. Furthermore, they have reemerged as important public health problems due to recent extreme weather events, human migration, urbanization and climate change [2, 8, 10, 12].

Leptospirosis typically causes two phases of infection: a mild anicteric phase in 80–90% of the patients and a classical icteric phase in the rest of the patients [2, 3, 13]. The anicteric form usually causes a mild febrile illness with minimal or no clinical manifestations, and the majority of patients display a nonspecific febrile syndrome undistinguishable from other causes of AFI [2, 3, 14]. However, a group of patients develop an icteric severe form, which include hepatic, pulmonary and acute renal failure known as Weill’s disease [13, 14]. In a similar way, most of the patients with DENV infection present with a broad clinical spectrum, ranging from a mild fever to classical dengue fever with hemorrhage and/or shock [6, 7].

The overlapping spectrum of signs and symptoms makes the diagnosis difficult, particularly in the acute phase of the disease [15–17]. The differential diagnosis of leptospirosis and dengue fever remains a major challenge for surveillance programs in resource-limited settings, as both have similar clinical profiles and seasonal onset [16]. Usually, DENV is thought to be the first probable diagnosis in patients with AFI in endemic areas. Moreover, the lack of symptom specificity, lack of appropriate diagnostic methods, and passive characteristics of the surveillance programs in affected regions may underestimate leptospirosis burden [16–18]. However, the diagnosis of one pathogen does not exclude the other, as co-infection between both have been described with a prevalence of 3.4% and 4.1% [10, 15].

The study of the pathogens responsible for AFI in Peru is crucial to understand their real impact in the population and to guide local clinicians to perform an accurate diagnosis. We hypothesize that many cases initially cataloged as dengue fever, may be in fact leptospirosis. Therefore, this study aimed to detect the presence leptospirosis in patients with AFI and a suspected diagnosis of dengue virus.

## Main text

### Methods

#### Study site

We performed a consecutive cross-sectional study in Piura, Peru between March and August of 2016 within eight urban primary health care centers from the “Regional Directive of Health of Piura”. The region of Piura is located in the northern coast of Peru, sharing boundaries to the north with Ecuador. Piura has an estimated population of 1,856,809 inhabitants.

#### Patients

A total of 276 patients were enrolled from March to August 2016 as part of the national dengue surveillance program. The inclusion criteria consisted of patients attending outpatient health centers with acute febrile illness (axillary temperature equal or greater than 38 °C in the previous 7 days) and a clinical suspicion of dengue fever according to the Peruvian Ministry of Health guidelines, which consist of AFI plus two of the following symptoms: arthralgias, myalgias, headache, retro ocular pain, lumbar pain and rash. Patients with an identifiable source of infection such as upper respiratory infection, urinary tract infection, among others were excluded.

#### Samples

One serum sample per patient was collected by using Vacuette^®^ TUBE Serum Separator Clot Activator (Vacuette, Greiner Bio-One, Kremsmünster, Austria). After collection, all samples were centrifuged at 3000 rpm for 5 min and the serum is stored at − 80 °C to perform molecular assays.

#### Detection of *Leptospira* IgM antibodies based-ELISA assay

Participants with a positive IgM ELISA sample, were considered positive for leptospirosis infection. Levels of antibodies were quantified using High Sensitivity Leptospira ELISA Kit (Abcam, United State). Each serum sample was run in duplicate, in accordance with the manufacturer’s instructions.

#### Real-time RT-PCR assay detection DENV serotypes with the TaqMan probe

RNA extraction was performed from 200 μL of the serum samples with the High Pure RNA Isolation Kit (Roche Applied Science, Mannheim, Germany), according to the manufacturer’s instructions. DENV amplification was performed using the Transcriptor High Fidelity cDNA Synthesis Kit (Roche Applied Science, Mannheim, Germany) and the primers, the probe and conditions were previously described [19]. All the procedure was performed in Light Cycler^®^ 2.0 Instrument and data was analyzed with the LightCycler^®^ Software 4.1 (Roche Diagnostic, Deutschland-Mannheim, Germany).

#### Ethics statement

The study protocol was approved by the Research Ethics Board of the *Hospital Regional Docente de Cajamarca*, Cajamarca, Peru. The samples were obtained in the context of the epidemiological/syndromic surveillance program according to the health directives of the National Center for Epidemiology, Disease Control and Prevention of the Ministry of Health of Peru.

#### Statistical analysis

The collected data were reported as frequencies and percentages. Chi square test was performed to estimate statistical difference between the variables, a value of P < 0.05 was considered significant. All analyses and figures were processed in the GraphPad Prism 9 program (San Diego, CA).

### Results

A total of 276 febrile patients with a clinical suspicion of dengue fever were studied from March to August 2016. Patients were grouped by ages, the majority of patients corresponded to the group between 20 and 44 years old with 33.3% cases, followed by the group between 5 and 19 years old with 26.5% the group between 45 and 59 years old and the one of 60+ were both 17.4%, respectively. There were no differences between gender with 143 (51.8%) female and 133 (48.2%) male patients (Table 1).

**Table 1:** Demographic characteristics in AFI patients with dengue, leptospirosis and co-infection

Characteristics	Total cases n (%)	Dengue n (%)	*Leptospira* n (%)	Co-infection n (%)	P-value
Age					
0–4	15 (5.4)	4 (4.6)	0 (0.0)	1 (6.3)	0.42
5–19	73 (26.5)	13 (14.9)	8 (25.8)	2 (12.5)	0.37
20–44	92 (33.3)	29 (33.3)	15 (48.4)	6 (37.5)	0.40
45–59	48 (17.4)	14 (16.7)	5 (16.1)	4 (25.0)	0.70
60+	48 (17.4)	24 (28.6)	3 (9.7)	3 (18.7)	0.09
Gender					
Male	133 (48.2)	42 (50.0)	12 (38.7)	9 (56.3)	0.44
Female	143 (51.8)	42 (50.0)	19 (61.3)	7 (43.7)	0.44
Total	276 (100.0)	84 (100.0)	31 (100.0)	16 (100.0)	
Positive cases (%)	47.5	30.4	11.2	5.9	
CI 95%	42.5–52.5	25.0–35.8	7.5–14.9	3.2–8.6	

**Fig. 1:**
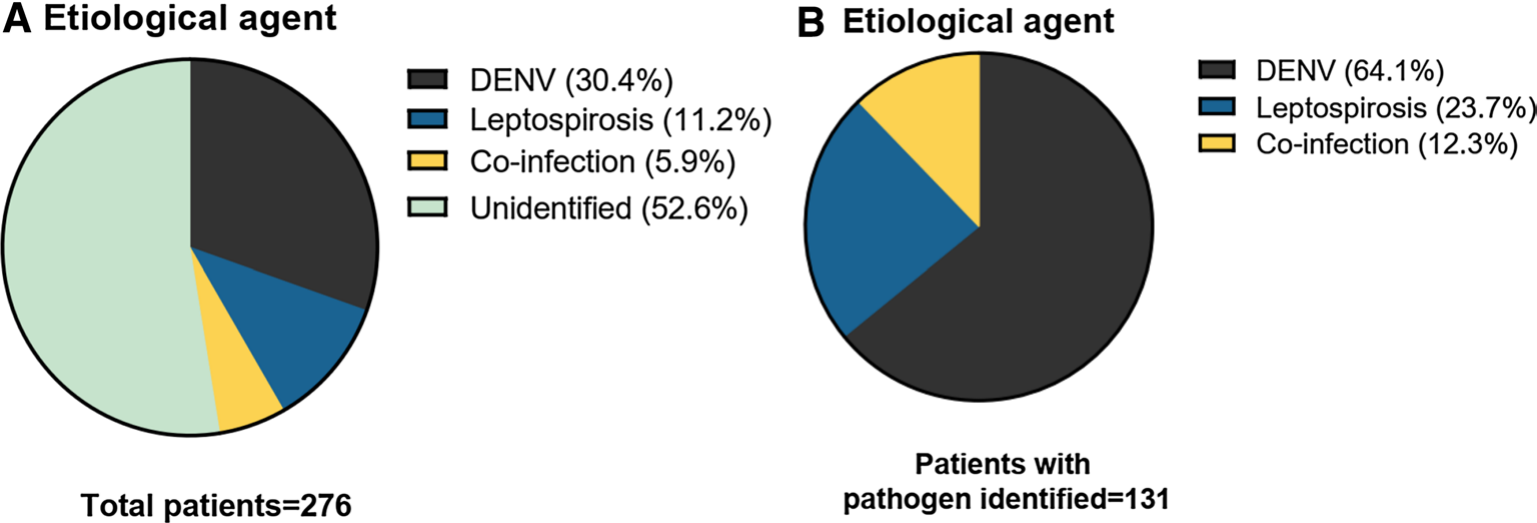
Frequency of etiological agents identified in patients with AFI. **A** Frequency of the etiological agents detected in the total population. **B** Distribution of the pathogens identified in the group of positive patients

We identified an etiological agent in 121 (47.5%) patients of all clinically diagnosed AFI and a suspected diagnosis of dengue fever. The majority of the patients were diagnosed with DENV accounting for 84 cases (30.4%), infection by only leptospirosis was detected in 31 patients (11.2%) and co-infection by both pathogens was observed in 16 (5.9%) of the patients (Fig. 1A). Of the total population, a pathogen could not be identified in 156 patients. Among the 121 patients with an etiological agent identified, the vast majority were indeed dengue infection with a 64%. However, leptospirosis and co-infections were detected in approximately a third of the patients with 24% and 12%, respectively (Fig. 1B).

In this study the most common clinical symptoms reported by all the patients were: headache 89.1%, myalgias 86.9% and arthralgias 82.9%. Patients infected only by dengue virus reported 85.1% myalgia, 83.9% headache and 81.6% arthralgia. In the case of patients with only leptospirosis infection the most frequent clinical symptoms were 96.7% headache, 90.3% arthralgias and 87.1% myalgias. Surprisingly, the 100% of the patients with co-infection reported headaches and myalgias, and 87.5% arthralgias (Table 2).

**Table 2:** Clinical characteristics in patients with dengue, *Leptospira* and co-infections

Clinical symptoms	Total cases		Dengue		*Leptospira*		Co-infection	
	n = 276	%	n = 84	%	n = 31	%	n = 16	%
Headache	246	89.1	73	83.9	30	96.7	16	100.0
Myalgia	240	86.9	74	85.1	27	87.1	16	100.0
Arthralgias	229	82.9	71	81.6	28	90.3	14	87.5
Retroocular pain	199	72.1	60	69.0	25	80.6	13	81.3
Hyporexia	177	64.1	45	51.7	21	67.7	11	68.8
Nauseas/vomiting	137	49.6	40	46.0	13	41.9	7	43.8
Low back pain	158	57.2	54	62.1	14	45.2	15	93.8
Odynophagia	105	38.0	27	31.0	11	35.5	5	31.3
Rash	60	21.7	16	18.4	9	29.0	1	6.3
Abdominal pain	14	5.1	3	3.4	1	3.2	1	6.3
Platelet decrease	6	2.2	1	1.1	0	0	1	6.3
Petechiae	10	3.6	1	1.1	1	3.2	1	6.3
Epistaxis	4	1.4	0	0	0	0	0	0
Gingivorrhagia	1	0.4	0	0	0	0	0	0
Hemoptisis	1	0.4	0	0	0	0	0	0
Chest pain	3	1.1	1	1.1	0	0	0	0
Altered mental status	1	0.4	1	1.1	0	0	0	0
Increase in hematocrit	5	1.8	1	1.1	0	0	0	0
Jaundice	1	0.4	0	0	0	0	1	6.3
Gynecorrhagia	1	0.4	0	0	1	3.2	0	0
Melaena	1	0.4	0	0	0	0	0	0
Decrease diuresis	1	0.4	0	0	0	0	0	0
Echimosis	1	0.4	0	0	0	0	0	0
Persistent vomiting	5	1.8	1	1.1	0	0	1	6.3
Hypothermia	3	1.1	0	0	0	0	0	0
Lipotimia	3	1.1	0	0	0	0	0	0
Hypotension	1	0.4	0	0	0	0	0	0
Chills	2	0.7	1	1.1	0	0	0	0
Cough	1	0.4	1	1.1	0	0	0	0

### Discussion

Acute febrile illness (AFI) encompasses a broad spectrum of infectious causes; however, it remains poorly characterized in tropical regions of the developing world [20–22]. Commonly, health care providers in developing countries find themselves in the need to apply syndrome-based treatment protocols, due to the lack of diagnostic tests [20, 21]. Dengue virus (DENV) and leptospirosis are among the most common causes of AFI [10, 15, 17, 20–22]. They often pose a diagnostic challenge for health care providers in tropical and subtropical regions; therefore, we performed the first study in northern Peru to characterize the burden of leptospirosis infection among patients seeking for medical care with a dengue-like illness.

We evaluated 276 patients in outpatient health centers for the presence of leptospirosis in patients with a probable diagnosis of DENV, in the context of the dengue epidemiological surveillance. An etiological agent was detected in 47.5% of the patients. Out of the total samples 30.4% tested positive for DENV as a single infectious agent by RT-PCR, 11.2% were positive for leptospirosis as a single infectious agent by detection of IgM ELISA and 5.9% were infected by both pathogens. We could highlight that accurate and reliable techniques for detection of the pathogens were performed, such as real time RT-PCR in the case of DENV and detection of IgM ELISA-based assay in acute sera of leptospirosis [6, 14].

We found an important frequency of leptospirosis among febrile patients with a probable diagnosis of dengue, with a frequency of 17.1% (11.2% as single infection and 5.9% as co-infection) in the total samples. Moreover, among the 121 patients in which an etiological agent was identified, at least a third of the patients had the diagnosis of leptospirosis. If fact, many regions in Peru are considered endemic and hyper-endemic for this disease, particular due to occupational exposure [23–25]. The largest study of leptospirosis in Peru was carried out between 1994 and 2004, identifying this pathogen in 18 of the 24 regions of Peru, being predominantly in the amazon [25]. However, most of the cases detected were severe and/or icteric forms of the disease, therefore, milder cases might have been left out. Also, a prevalence of leptospirosis ranging from 11.1 to 36.6% have been reported in patients with AFI in Peru [26].

Previous studies have also reported the detection of leptospirosis among patients with dengue-like symptoms in other settings. Dircio-Montes, et al. established the prevalence of leptospirosis in patients with an initial diagnosis of dengue, they concluded that at least a sixth part of the cases were leptospirosis infection and should have been treated with antibiotics [27]. Libraty et al. also reported that leptospirosis accounted for 19% of dengue suspected patients that tested negative for DENV [18]. Other studies have reported a lower prevalence of leptospirosis among dengue suspected patients ranging from 5 to 7.35% [28, 29]. Moreover, it has been reported that many leptospirosis cases may go under recognized during dengue outbreaks [30, 31].

On the other hand, we could not determine the etiological agent in half of the patients studied. The detection of two pathogens of interest was evaluated, however, other emerging and re-emerging infectious pathogens causative of AFI have become more prevalent in our country, such as Zika virus, Chikungunya virus, Mayaro virus, Oropouche virus, *Rickettsia* spp, *Bartonella* spp, among others [32–34].

Clinical characteristics were also evaluated regarding the etiology of the infection. We could observe the presence of unspecific symptoms among all groups. The three groups of patients reported similar frequencies of headache, myalgias and arthralgias as predominant symptoms accompanying fever, we could observe the overlapping signs and symptoms that patients with leptospirosis and dengue may present. This highlights the need for available diagnostic tests to accurate diagnose leptospirosis in patients with probable dengue diagnosis. However, we observed that 100% of the patients with co-infections reported headache and myalgia, also a high frequency of arthralgias. Also, the only case of jaundice reported in our study correspond to this group. This is in agreement with previous studies, which have reported fatal cases and severe disease when both pathogens are present [35].

In conclusion, we found that an etiological diagnosis could be achieved in 47.5% of the total cases. Leptospirosis was identified in 17.1% (11.2% as single infection and 5.9% as co-infection) of the patients with suspected dengue diagnosis. Accurate and early diagnostic tests are crucial as clinical signs and symptoms may be overlapping. It is of great importance to differentiate these two pathogens as patients with leptospirosis may benefit from antibiotic use.

### Limitations

Our main limitation is that we could not identify the etiological agent responsible for the febrile illness in nearly half of the patients and other pathogens could be involved, however, we were focused on two pathogens of interest. Another limitation is that not all of the patients with AFI could attend to outpatient health centers and we could have missed more severe cases.

## Data Availability

Abstraction format used in the study and dataset are available and accessible from the corresponding author upon request in the link: https://figshare.com/s/a21c004df98dce729476

## Abbreviations

AFI: Acute febrile illness
DENV: Dengue virus
RT-PCR: Reverse transcription polymerase chain reaction
